# Tissue Specific Labeling in Proteomics

**DOI:** 10.3390/proteomes5030017

**Published:** 2017-07-18

**Authors:** Evelyn Ramberger, Gunnar Dittmar

**Affiliations:** 1 Mass-Spectrometry Core Unit, Max Delbrück Center for Molecular Medicine, 13125 Berlin, Germany; evelyn.ramberger@mdc-berlin.de; 2 Berlin School of Integrative Oncology (BSIO), Charité-Universitätsmedizin Berlin, 13353 Berlin, Germany; 3 Proteome and Genome Research Laboratory, Luxembourg Institute of Health, 1272 Strassen, Luxembourg

**Keywords:** quantitative proteomics, stable isotope labeling, tissue, amino acid analog, biotinylation, bioorthogonal, multicellular, SILAC, APEX, BioID

## Abstract

Mass spectrometry-based proteomics is a powerful tool for identifying and quantifying proteins in biological samples. While it is routinely used for the characterization of simple cell line systems, the analysis of the cell specific proteome in multicellular organisms and tissues poses a significant challenge. Isolating a subset of cells from tissues requires mechanical and biochemical separation or sorting, a process which can alter cellular signaling, and thus, the composition of the proteome. Recently, several approaches for cell selective labeling of proteins, that include bioorthogonal amino acids, biotinylating enzymes, and genetic tools, have been developed. These tools facilitate the selective labeling of proteins, their interactome, or of specific cell types within a tissue or an organism, while avoiding the difficult and contamination-prone biochemical separation of cells from the tissue. In this review, we give an overview of existing techniques and their application in cell culture models and whole animals.

## 1. Introduction

Higher organisms are composed of different cells, allowing the individual cell to specialize, and thus become more efficient in performing specific tasks. The coordination of biological functions requires complex signaling events, and consequently, the proteomes of cells in a heterogeneous community will differ from the proteomes of cells that are kept in monocultures. The analysis of these complex intercellular communication systems poses a significant challenge to modern cell biology and biochemistry. Only recently, methods have been developed which are capable of addressing this issue. The use of engineered enzymes and bioorthogonal amino acids, in combination with other chemical compounds, now allows the analysis of multicellular environments in a cell or tissue specific manner. In this review, we are focusing on the methods that have been successfully applied in the field of proteomics.

## 2. Mass Spectrometry-Based Proteomics

Over the last few years, mass spectrometry-based proteomics has become one of the main techniques for the analysis of proteomes on a large scale. The development of new instruments, and in parallel, new procedures for sample preparation, have pushed the technique to new horizons, with the depth of the analysis and throughput constantly increasing. The first comprehensive analysis of the yeast proteome was reported in 2008 [1], and with state-of-the art technology, this benchmark can now be reached with a very limited analysis time of one hour [2]. The complete analysis of the human proteome, although not reached yet, is not far away, as current literature reports the identification of around 10,000 proteins in HeLa cells [3]. 

**Figure 1:**
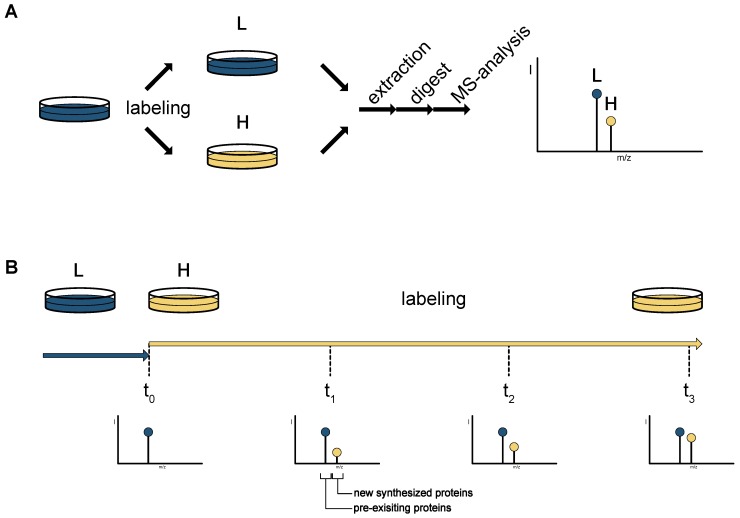
Amino acid based labeling techniques. (**A**) SILAC labeling. Proteins are metabolically labeled in cell culture with isotope-coded amino acids (typically lysine or arginine, light (L)/heavy (H)). All unlabeled lysine and arginine is removed and replaced with the isotope labeled counterpart (yellow dish). The two cultures are now combined and jointly prepared for mass spectrometric analysis. In the mass spectra, the isotope labeled peptides give rise to a double peak–SILAC pair (L and H), from which the peptide and protein abundance ratios can be inferred. (**B**) Dynamic labeling with SILAC amino acids. At the start point of the experiment (t_0_) the media is switched from unlabeled to labeled. All proteins synthesized after this time point are isotopically labeled. This corresponds to an increasing peak in the mass spectra for the labeled peptides (yellow peaks). Samples are collected at different time points, allowing the analysis of the changes in protein synthesis.

Quantification of changes between proteomes is one of the main goals and challenges addressed by mass spectrometry-based proteomics. Several isotope-based labeling techniques have been developed and utilized in the past. While some of them introduce the label using chemical modification, the use of stable isotope labeling by amino acids in cell culture (SILAC) has been proven to be a very useful and robust method for the labeling of proteins in cell culture [4]. The basis for this labeling technique is the stepwise replacement of essential or conditionally essential amino acids (typically lysine and arginine) by an isotope-labeled analog in cell culture (Figure 1—labeling techniques). The labeled cells can then be combined prior to lysis, and processed together. The resulting light and heavy peptides generate characteristic mass pairs in the precursor mass detection, and the direct comparison of the two peptide intensities enables relative quantification of the peptides and the proteins they originate from. SILAC is widely used in cell culture, but is also applicable for labeling whole model organisms, including mammals [1,5,6]

Although very robust, this method has significant shortcomings in the analysis of proteomes from different cell types in co-culture. Pre-labeled cells, when transferred to media with another label, dilute the signal as they divide and synthesize new proteins, but for short incubations, this method has been successfully used [7,8]. In the trans-SILAC approach, differentially labeled cells are co-cultivated for some time and subsequently sorted back into homogeneous populations with FACS (Fluorescence-activated cell sorting). The two populations are then analyzed for proteins which have been transferred (trans-SILAC) between the different cell types during co-culturing [8]. 

To achieve complete labeling of the proteome, cells should be cultivated for at least five doubling times in SILAC media. Differentially labeled proteomes (“heavy”, “medium-heavy” and/or “light”) can be directly combined and compared with a single analysis, but it is also possible to expose cells to SILAC media for a limited amount of time (pulsed SILAC, Figure 1B) and thereby investigate changes in protein synthesis [9,10,11]. Due to the lack of enrichment strategies for SILAC, the low abundance of newly synthesized proteins hampers identification and quantification in experiments with short pulses. If the SILAC pulse is continued for a prolonged amount of time, the method is sometimes also referred to as dynamic SILAC, which is focused on the quantification of protein turnover after a certain stimulus [12], and has also been successfully used in mice [13]. 

## 3. Bioorthogonal Amino Acids 

There are several bioorthogonal amino acids available for metabolic labeling. These amino acid analogues are either coupled to tRNA by endogenous tRNA synthetases [14,15,16,17], or by modified tRNAs synthetases [18,19,20,21] that need to be genetically transferred to the model organism or cell line of choice. The unnatural amino acids (UAA) often contain a functional group that can be linked to an affinity tag for enrichment, or a fluorescent probe for imaging. Bioorthogonal non-conanonical amino acid labeling (BONCAT) overcomes the limitations of SILAC regarding cell specificity and possibility for enrichment. 

## 4. Enrichment Using High Affinity or Covalent Binding

Classical biochemical and cell-biological preparation methods allow the isolation of subcellular compartments and cells of a particular cell type, the method spectrum ranging from differential centrifugation and general chromatography techniques to affinity enrichment. However, all these methods are hampered by the same challenges: the significant amount of starting material needed, and the high level of cross contamination with other parts of the cell. For cell biological enrichment techniques, the problem of cross contamination is highly dependent on the method, and while FACS allows a rather high purity, affinity purification based techniques can include significant amounts of non-targeted cells. FACS generates high quality samples, but the protein amounts from cells that can be sorted are rather small, and require significant sorting times at the machine. If whole tissues are analyzed, the technique requires dissociation of the tissue and generation of a single cell suspension, which by itself can pose a challenge and induce proteomic changes in the analyzed cells. 

**Figure 2:**
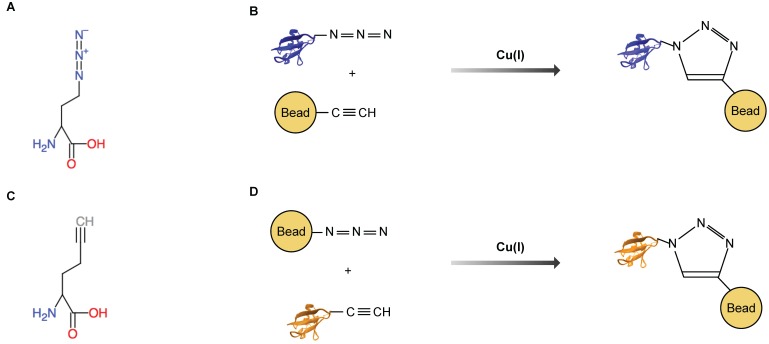
Covalent binding of proteins using bioorthogonal amino acids. (**A**) Azidohomoalanine (AHA). (**B**) AHA labeled proteins are bound by cyclo-addition in the presence of Cu(I) to a resin containing an alkyne residue. (**C**) homopropargylglycine (HPG). (**D**) HPG labeled proteins are bound by cyclo-addition to a resin containing an azide group.

An alternative approach is the introduction of high affinity binding groups at the cellular or subcellular level, with the small molecule biotin often being the tag of choice. As a small molecule, it is able to penetrate tissue and diffuses easily to different parts of the cell. Biotin ligating enzymes link biotin to the target protein, and the labeled protein can then be isolated using the extremely high affinity of avidin (K_d_ ≈ 10^−15^ mol/L) or streptavidin beads (K_d_ ≈ 10^−14^ mol/L) for biotin. Another possibility to bind labeled proteins specifically is a covalent bond. In that case, a specific amino acid analogue has to be introduced into the protein. These modified amino acids contain either an azide or an alkyne group as a side chain. One of the special properties of azides and alkynes is the ability to form a ring structure (cyclo-addition) in the presence of a catalyst (click chemistry, Figure 2). As shown in Figure 2, the formation of this covalent bond can be induced after the denaturing lysis of cells and tissues, binding the proteins covalently to the resin of choice through an efficient, high yield reaction [22]. Covalent capture permits much harsher washing protocols, and thereby minimizes the background.

## 5. Azidohomoalanine and Homopropargylglycine

The methionine analogues azidohomoalanine (AHA) and homopropargylglycine (HPG) are able to bind to the endogenous methionyl-tRNA synthetase (MetRS), and are thus incorporated into proteins in the place of methionine [14,15,16]. AHA and HPG carry an azide or an alkyne group, respectively that enables covalent binding to a resin bound tag for enrichment or a fluorescent tag for imaging via click chemistry. The affinity of AHA and HPG to MetRS is 390–500 fold lower than the one of methionine, and it is advisable to perform the labeling in methionine free media after a methionine depletion step [23]. However, it has been shown that a 24 h long AHA pulse in methionine-free media induces substantial protein abundance differences. Supplementing media with both AHA and methionine in a ratio of 30:1 minimized the effect, but also compromised the number of identified proteins [24]. Despite the lower affinity of AHA and HPG to MetRS compared to methionine, several studies have demonstrated that these UAAs are also incorporated into proteins in vivo, in zebrafish, frog, and mice, and do not affect embryonic development [17,25,26,27]. The possibility for enrichment makes short labeling times (pulses) feasible, and predisposes the technique for investigating newly synthesized proteins and proteome dynamics [28,29,30]. Labeling with AHA and SILAC has also been combined to analyze newly synthesized and secreted proteins in activated macrophages [31], and to measure translation profiles in brain slices [32]. 

## 6. Modified Aminoacyl tRNA Synthetases

Cell selective tagging of proteins with UAAs has become possible by the combination of engineered tRNA synthetases, and UAAs that are recognized by the mutant tRNA synthetases but are only poor substrates for endogenous enzymes. In 2006, the Tirell lab reported a mutant *Escherichia coli* MetRS that is capable of coupling the methionine surrogate azidonorleucine to methionyl-tRNA [19]. Importantly, the azide group of azidonorleucine enables enrichment of labeled proteins by click chemistry. Azidonorleucine incorporation into proteins is limited to cells that express the mutant synthetase, and the approach has been used by several studies to differentially label proteins originating from pathogens and host cells during bacterial infection [18,33,34]. The mutant MetRS approach was further developed into a two-step activatable system, based on the construction of a split MetRS that generates a translational output only in cells in which inputs from two promoters are on at the same time, enabling fine tuning of the labeling [35]. A mutant MetRS was also introduced into mammalian cells, but drawbacks of the method are that only N-terminal methionines are labeled, and that the mutant MetRS activated methionine four times faster than azidonorleucine [36,37].

Recently, labeling with azidonorleucine has been adapted for usage in *Drosophila melanogaster* [20], by mutating a single amino acid residue within the MetRS binding pocket from *D. melanogaster*, and expressing the mutated enzyme under cell specific promoters. Importantly, internal methionine residues can also be labeled with this method. The investigators note that prolonged labeling throughout all developmental stages affected larval growth, but no effect was observed when labeling was restricted to a period of 48 h [20]. 

Cell specific labeling with UAAs is also possible in *Caenorhabditis elegans*. Using a mutant phenylalanyl-tRNA synthetase allows the charging of phenylalanyl-tRNA with the noncanonical amino acid p-azido-l-phenylalanine. The mutant enzyme is selective for p-azido-l-phenylalanine, and starvation of phenylalanine is not required. Cell selective expression of the synthetase and reacting the azide group of the UAA with a fluorescent or enrichment tag (click chemistry) enable enrichment or visualization of tissue specific proteins [38]. 

In stochastic orthogonal recoding of translation with chemoselective modification (SORT-M), an orthogonal aminoacyl-tRNA synthetase/tRNA pair is introduced into cells. The anticodon of the orthogonal tRNA can be engineered to recognize different sets of sense codons, and there is no competition at the active site of the synthetase by natural occurring amino acids. It is therefore possible to perform the labeling in rich media or with a normal diet supplemented with the UAA. Labeling is feasible in a variety of cell types and organisms, and did not affect oocyte development in *D. melanogaster* [21]. SORT-M was successfully combined with enrichment of tagged proteins (SORT-E) by reacting a cyclopropene-containing bioorthogonal amino acid and a tetrazine-biotin probe with a cleavable linker. The results demonstrate that SORT-E is applicable for cell specific proteomics, but the authors note that tagging at different codons led to overlapping, but distinct, sets of proteomes, and suggest tagging at more than one codon for increased proteome coverage [39]. SORT has already been applied in the brain of live mice, and can be expanded to contain UAAs that mimic a posttranslational modification [40,41]. 

A similar approach can be used to isolate specified proteins of interest without the need for an enrichment tag that could interfere with secondary structure and function of the protein. In click-MS, a construct containing the protein of interest with an amber (TAG) stop codon [42] is introduced into the host cell together with a modified tRNA and tRNA synthetase. The UAA p-azido-l-phenylalanine is then selectively incorporated into the protein at the place of the amber codon, and the protein is subsequently enriched via click chemistry [43]. 

## 7. Isotopic Labeling of Amino Acid Precursors (CTAP and NANCAT) 

Cell selective labeling with amino acid precursors (CTAP) avoids the problems that might arise from structural differences of bioorthogonal amino acids. Lysine is an essential amino acid for vertebrate cells, but can be synthesized by plants, bacteria, and lower eukaryotic organisms. By genetic incorporation of lysine synthesizing enzymes (lysine racemase or diaminopimelate decarboxylase), and isotope labeling of their substrates (d-lysine or 2,6-diaminopimelic acid, respectively), heavy lysine labeling of cells that express the transgene can be achieved [44]. Tape et al. have optimized the original protocol and showed that labeling efficiency is comparable to SILAC [45]. CTAP can be used to differentially label proteins from distinct cell types in co-culture, and has been combined with phosphoproteomics to dissect *KRAS* dependent signaling between pancreatic tumor and stromal cells [46].

Nitrilase-activatable non-canonical amino acid tagging (NANCAT) exploits a similar strategy that is based on the enzymatic conversion of nitrile-substituted precursors to their corresponding non-canonical amino acids AHA or HPG [47]. Labeled proteins can be enriched with click chemistry, but unlike CTAP, the NANCAT system does not allow the introduction of two different labels for cells in co-culture. 

## 8. Genetic Labeling

### 8.1. O-Propargyl-Purocmycin Labeling (OP-Puro)

OP-Puro labeling has originally been developed for fluorescence imaging of protein synthesis. The puromycin analogue OP-Puro binds to the acceptor site of translating ribosomes and forms a covalent bond between the C-terminus of the nascent polypeptide chain and the primary amine group of OP-Puro. Labeled newly synthesized proteins can be enriched, or visualized, by reacting the alkyne group of OP-Puro with a biotin-azide or with a fluorescent azide respectively [48]. The technique was further modified by adding a labile blocking group to OP-Puro, which can be removed by the *E. coli* enzyme, penicillin G acylase. Cell selective labeling and enrichment of actively translated polypeptides can be accomplished by targeted expression of the enzyme. This technique requires very short labeling times, and provides a snapshot of newly synthesized proteins in a subset of cells at a certain time point [49]. 

### 8.2. Specific Labeling with Biotin

**Figure 3:**
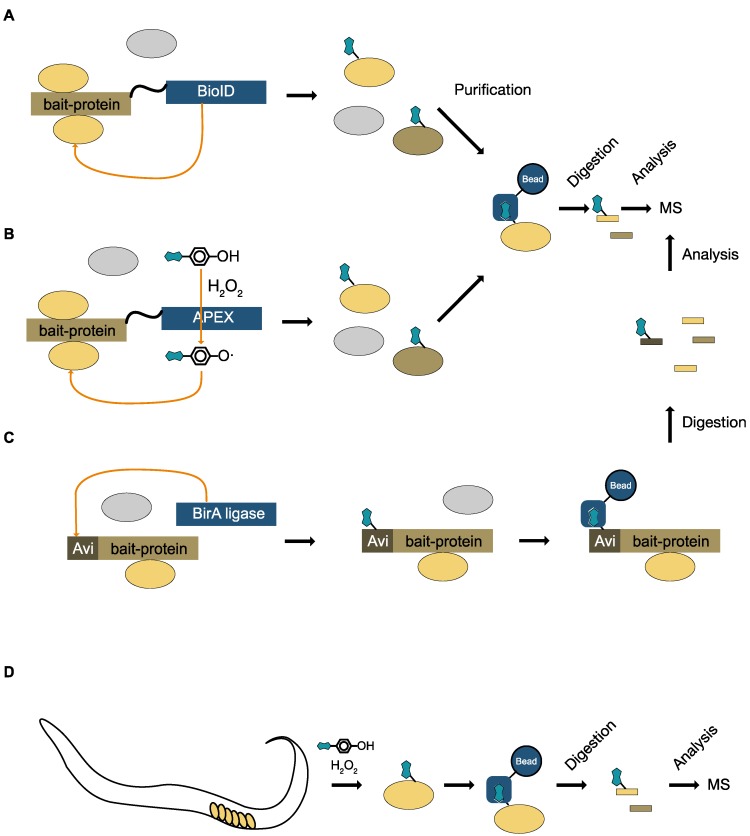
Biotin based in vivo labeling techniques. (**A**) BioID method. The protein of interest is expressed as a fusion protein with the BioID biotin ligase. The BioID ligase transfers biotin to interacting proteins in proximity to the protein of interest. Using high affinity binding resins, the biotinylated proteins are isolated, digested, and analyzed by mass spectrometry. (**B**) APEX. The protein of interest is expressed as a fusion protein with the APEX enzyme. In the presence of biotin–phenol and H_2_O_2_, the APEX enzyme transfers biotin to the interacting proteins. The biotinylated proteins are isolated and analyzed. (**C**) Avi- or BirA-tag. The protein of interest is fused to the Avi tag. The coexpressed BirA biotin ligase transfers biotin to the Avi-tag. High affinity chromatography is used to isolate the in vivo biotinylated protein of interest and its interactors. The protein of interest and the interactors are digested and analyzed by mass spectrometry. (**D**) Labeling of a specific cell type in *C. elegans*. By expressing the APEX enzyme under the control of a tissue specific promoter, only the proteins in a subpopulation of cells are labeled with biotin. After extraction of the whole proteome, labeled proteins can be easily purified and analyzed.

In vivo labeling of specific proteins is a powerful tool to monitor the fate of proteins, or changes to their localization. One system that allows the tissue specific labeling of individual proteins in different tissues is the expression of the *E. coli* derived biotin-ligase, BirA, in combination with the tagging of the gene of interest with a specific amino acid sequence, the so called BirA- or Avi-tag. The BirA ligase recognizes the tag and adds a biotin onto the tag (Figure 3C), which can then be used for isolation of the protein, or labeling for microscopy. If the expression of the biotin ligase is restricted to specific tissues, the BirA system can be used to identify tissue specific complex formation. Using this technique, the neuron specific interaction of DLG-1 and MAPH-1.1 in *C. elegans* was identified in a study comparing complex composition in four different cell types in vivo [50].

### 8.3. Unspecific Labeling with Biotin

The promiscuous biotin ligase from *E. coli* has been successfully used in protein–protein interaction studies (BioID [51]). For this, the biotin ligase has been fused to the protein of interest and expressed in the cell, leading to the biotinylation of interacting proteins that are physically close to the biotin ligase. The biotinylated proteins are then isolated by avidin-based precipitation techniques and identified by mass spectrometry (Figure 3A). Since this approach is based on a genetic construct that generates a fusion protein with the biotin ligase, it can be used for the specific labeling of interacting proteins in selected tissues. This is achieved by combining the expression of the fusion protein with a tissue specific promoter, allowing only the expression in a subset of cells in the tissue. After labeling of the interacting partners, the proteins of the tissue are extracted by standard techniques, and the biotinylation is detected by mass spectrometry [52]. Recently, an improved version of the BioID ligase has been developed, which is smaller in size, and more efficient in transferring biotin to its targets [53]. However, labeling with BioID remains rather slow, requiring labeling times of around 24 h. 

An alternative method for labeling interacting proteins, or proteins in a particular subcellular compartment, is the use of APEX, an engineered enzyme derived from soy or pea ascorbate peroxidase [54,55]. The APEX enzyme is engineered to be targeted to a specific compartment [54] or a specific cell type. The live cells are then treated for a very short time (~1 min) with hydrogen peroxide in the presence of biotin–phenol. This generates highly reactive biotin–phenol radicals that react with proteins in their vicinity. Since the reaction is driven by intermediate radicals, the labeling reaction is very rapid. The biotin tag can again be used for the purification of the labeled proteins and subsequent mass spectrometric analysis (Figure 3B). Since the original APEX has some limitations, in terms of stability to heat and a reducing environment, an improved APEX2 enzyme has been engineered [56]. The specific expression of mitochondrion-localized APEX in different fly tissues was used to compare the mitochondrial composition in different tissues [57], or to analyze the specific proteomes in different tissues of *C. elegans* [58] (Figure 3D). 

### 8.4. GFP-Labeling of a Subpopulation of Cells Combined with FACS 

Expression of GFP under a cell specific reporter, followed by FACS sorting of the cells, enables enrichment of the respective cell type or tissue for proteomics. Palma et al. report the colon stem cell specific proteome from a recombinant mice strain that expresses GFP under the lineage specific Lgr5 promoter [59]. A similar approach is also applied in analyzing the proteome of sub-populations of prokaryotic cultures, and is reviewed here [60].

## 9. Analysis of Cell and Tissue Specific Signaling

### 9.1. Tyrosine Signaling

In order to address the signaling changes in response to ephrin (Eph) receptor ligand interaction, the laboratory of Tony Pawson used a combination of SILAC and cells expressing either Eph or the receptor (quantitative analysis of Bidirectional Signaling—qBidS). Cells expressing Eph were labeled with one type of label, while the receptor expressing cells were labeled with the other type of SILAC label. After incubating the two populations together for 10 min, the cells were lysed, and phospho-tyrosine peptides isolated and analyzed by mass spectrometry. The SILAC label could trace the signal back to the either the Eph or Eph-receptor expressing cells, allowing the construction of a specific signaling network for both sides of a cell-to-cell signaling system [7]. 

### 9.2. Bio-Ubiquitin

**Figure 4:**
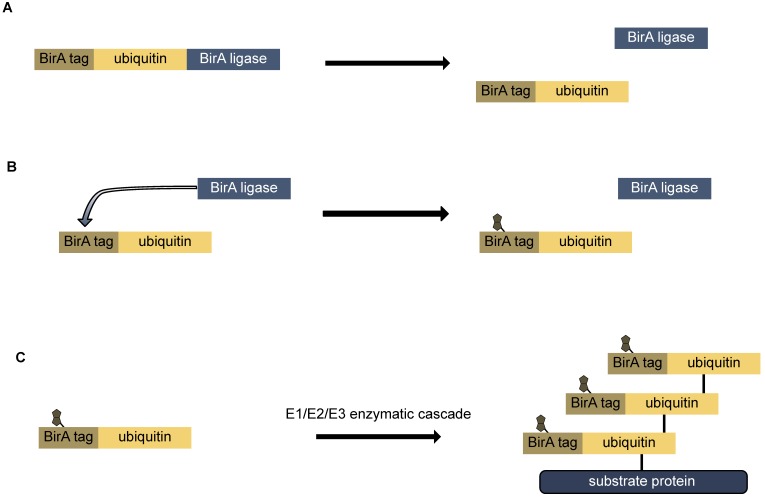
Analysis of ubiquitin signaling using bio-ubiquitin. (**A**) The ubiquitin-biotin-ligase fusion protein is expressed in the cells of interest. The tagged ubiquitin and the biotin-ligase are processed co-translationally. (**B**) The birA-ligase transfers a biotin moiety to the birA tag. (**C**) The in vivo labeled bio-ubiquitin is recognized by the E1/E2/E3 conjugation cascade and transferred to the substrate protein. Additional ubiquitin-moieties are added to the initial ubiquitin forming a poly-ubiquitin-chain.

An elegant example for the use of tissue specific promoters for tissue specific labeling, is the use of bio-Ubiquitin in ubiquitination assays. Ubiquitin is a signaling molecule which is involved in different types of cellular signaling, including specific degradation, bulk degradation, stabilization of proteins, or DNA repair. Like other post-translational modifications, the labeling of substrate proteins is performed by an enzymatic cascade, which uses ATP to catalyze the covalent attachment of the C-terminus of ubiquitin to the amino group of lysine side chain, or the N-terminus of a protein [61]. Ubiquitin itself is a protein, and thus has to be synthesized prior to its use in the cell. Ubiquitin is expressed in the form of several N-terminal fusion proteins. These fusion proteins are proteolytically processed, co-translationally creating the mature ubiquitin protein, which is recognized by the conjugating enzymatic cascade. For the tissue-specific detection of ubiquitin labeling, the precursor of ubiquitin is fused to a BirA tag (Avi tag). The sequence of the BirA tag is recognized by the sequence specific biotin ligase BirA, and biotinylated (Figure 4). By combining the expression of the precursor and the ligase with a tissue specific promoter, the ubiquitination reaction can only occur in selected tissues. After a biotin -specific isolation, bio-ubiquitin modified proteins can be identified by mass spectrometry [62,63,64,65]. An alternative approach has recently been developed using an N-terminally tagged ubiquitin in combination with transcriptional repression of two of the four ubiquitin genes (StUbEx). This allowed an 80% replacement of the cellular ubiquitin with the tagged version, but had some impact on the transcription on the UBA52 ubiquitin gene, which is also responsible for the production of the ribosomal protein L40 [66].

## 10. Monitoring Signaling for Specific Enzymes

The identification of enzyme targets is a challenge by itself, and despite the fact that phosphorylation is one of the best studied post-translational modifications, the substrates of most human kinases are still unknown. One approach addressing this issue is the use of engineered kinases, which accept the ATP analogue adenosine-5′-γ-thiophosphate (ATPγS) instead of ATP. Expression of the modified enzyme leads to target proteins carrying a thio-phospho-group, which in turn can be used for enrichment by forming a covalent bond with an iodine activated resin. In subsequent steps the isolated proteins are identified by mass spectrometry. This method has been successfully used in several studies identifying substrates of the CDK2 or Clk1-cyclin B complex [67,68].

A similar approach has been developed for the identification of protein-methyltransferase substrates. These enzymes need S-adenosyl-l-methionine (SAM) as a co-factor, which provides the methyl group for the methylation reaction. Here, the binding pocket for SAM is mutated to accept a SAM analogue. Using this technique, methylation targets for GLP1/KMT1D, G9a/KMT1C [69] and PRMT3 [70] have been identified.

## 11. Summary and Outlook

Over the last decade, a number of proteomic labeling techniques have been developed, that range from metabolic labeling with bioorthogonal amino acids, to enzyme-driven modifications (summarized in Table 1). When choosing a method for protein labeling in vivo, several considerations regarding possibility for enrichment, cell specificity, and off-target effects, have to be made. Mechanical separation and sorting of a specific cell type or tissue is straightforward, but no temporal information is obtained, and the sorting procedure itself can induce proteomic changes. The SILAC methodology is well established for labeling proteins in cultured cells and whole organisms, but the lack of cell specificity and enrichment are limitations of the technique. The bioorthogonal amino acids AHA and HPG can also be utilized by all cell types, but the functional groups of both amino acid analogs allow the downstream enrichment of labeled proteins. This enables very short labeling times and the analysis of newly synthesized proteins. Cell specificity of labeling with bioorthogonal amino acids is achieved by mutated tRNA-synthetases that are engineered to recognize modified amino acids, but this requires genetic modification of the model system. The same is true for the SORT approach, where two constructs have to be transferred (engineered tRNA and tRNA synthetase). Advantages of SORT are that labeling can be extended to any codon, and the speed of the enrichment strategy. When labeling with bioorthogonal amino acids, side-effects arising from structural differences of the bioorthogonal amino acids are possible, and in addition, starvation of essential amino acids can be required. CTAP circumvents these problems by introducing lysine-synthesizing enzymes that metabolize isotope labeled lysine precursors into heavy labeled l-lysine. 

Nascent polypeptides can be labeled with OP-Puro, but the technique has not been widely applied for proteomics yet. The technique requires genetic transfer of the *E. coli* enzyme G acylase, and provides a snapshot of translation at a certain time point. 

If labeling of only a subpopulation of proteins in a cell is desired, labeling with a biotinylating enzyme is the method of choice. Labeling with biotin can either be restricted to a specific protein (BirA enzyme only biotinylates proteins with an Avi tag) or be applied to all proteins in vicinity to the enzyme (promiscuous biotin ligase or APEX). In contrast to BioID proximity labeling, labeling with APEX is very fast, and is also applicable for the labeling of subcellular compartments. For biotin labeling, genetic modification of the model system is necessary, and care should be taken in choosing a suitable promoter in order to avoid issues arising from overexpression of the fusion protein. Biotinylation is a modification that also occurs naturally in vivo, which can create a certain level of background. 

Tissue specific labeling techniques can also be used to investigate signaling pathways (qBidS) and post-translational modifications (ubiquitination, methylation, and phosphorylation). In contrast to bio-ubiquitination assays, where all ubiquitinated proteins are enriched through affinity purification of biotinylated ubiquitin, labeling of methylation and phosphorylation sites enables the identification of kinase and methylase targets. This is achieved by engineering modified enzymes that accept tagged ATP or SAM analogues, respectively.

The utilization of selective proteome labeling techniques will push the biochemical characterization of heterogeneous cellular environments to new limits, and help to unravel the complicated signaling events between different cells types. In the last years, cell-specific labeling has moved from cell culture to whole model organisms, including mammalian systems. 

## Figures and Tables

**Table 1:** Labeling Techniques.

Method	Label	Cell Specific?	Genetic Modification Necessary	Already Applied in	Enrichment of Labeled Proteins Possible	Time Scale and Applications	References
Stable isotope labeling of amino acids in cell culture (SILAC)	heavy isotope containing amino acids	no	no	wide range of cell lines and model organisms	no	5 doubling times to achieve complete labeling of a proteome. Pulsed labeling possible but number of identifications is compromised	[1,4,5,6,7,8,9,10,11,12,13]
Bioorthogonal labeling of amino acids in cell culture (BONCAT)	methione analogues AHA or HPG	no	no	wide range of cell lines and model organisms	yes (covalent capture with click chemistry)	short pulses (down to minutes) and subsequent enrichment of newly synthesized proteins. Prolonged labeling possible	[14,15,16,17,23,24,25,26,27,28,29,30,31,32]
Cell specific BONCAT	biorthogonal amino acids that require a modified tRNA sythetase (azidonorleucine or p-azido-l-phenylalanine)	yes	yes (mutated tRNA synthetase)	cell lines, worm, fly	yes (covalent capture with click chemistry)	short pulses (down to minutes) and subsequent enrichment of newly synthesized proteins. Prolonged labeling is possible but dependent on the system side effects are possible	[18,19,20,33,34,35,36,37]
Stochastic orthogonal recoding of translation (SORT)	bioorthogonal amino acid in combination with an orthogonal tRNA and tRNA synthetase	yes	yes (mutated tRNA synthetase and tRNA)	cell lines, fly, mouse brain	yes (covalent capture with click chemistry)	short pulses (down to minutes) and subsequent enrichment of newly synthesized proteins. Prolonged labeling is possible. Many codons can be tagged	[21,39,40,41]
O-propargyl-purocmycin labeling (OP-Puro)	Puromycin analogue (OP-Puro) binding to nascent polypeptides	yes	yes (penicillin G acylase )	cell lines	yes (covalent capture with click chemistry)	very short labeling (minutes). Provides a snapshot of actively translated proteins in a cell	[48,49]
isotopic labeling of amino acid precursors (CTAP)	heavy isotope containing lysine	yes	yes (lysine synthesizing enzymes)	cell lines	no	labeling comparable to SILAC. Cell specific labeling of cells in co-culture	[44,45,46]
GFP -labeling and sorting	GFP	yes	yes (GFP)	cell lines, unicellular organisms, mouse	sorting of labeled cells with FACS	steady state proteome of a subpopulation of cells	[59,60]
proximity-dependent biotin identification with a promiscous biotin ligase (BioID)	biotin	yes	yes (promiscous biotin ligase fused to protein of interest)	cell lines, unicellular organisms	yes (affinity purification with streptavidin)	proximity labeling of interacting proteins	[51,52,53]
biotinylation with sequence specific biotin ligase BirA	biotin	yes	yes (BirA and Avi tagged protein of interest)	cell lines, wide range of model organisms	yes (affinity purification with streptavidin)	biotinylation of tagged proteins only in cells expressing BirA. Purification of interacting proteins	[50]
	biotin-ubiquitin	yes	yes (Avi tagged Ubiquitin in fusion with BirA ligase)	cell lines, fly, mouse	yes (affinity purification with streptavidin)	biotinylation of ubiquitin and enrichment of ubiquitinated proteins	[62,63,64,65]
labeling with an engineered ascorbate peroxidase (APEX)	biotin phenol	yes	yes (APEX)	cell lines, fly, worm	yes (affinity purification with streptavidin)	proximity labeling of interacting proteins or cellular compartment specific proteins	[54,55,56,57,58]
